# Giant insulinoma: report of a case and review of published reports

**DOI:** 10.1186/s40792-016-0265-z

**Published:** 2016-11-19

**Authors:** Kazumitsu Ueda, Tetsuro Taira, Hiroyuki Hakoda, Shoko Nakata, Shinya Okata, Takeshi Nagai, Shigeo Aoki, Hideyuki Mishima, Akihiko Sako, Tsunehiko Maruyama, Minoru Okumura

**Affiliations:** Department of Surgery, Hitachi General Hospital, 2-1-1 Jonan-cho, Hitachi, Ibaraki 317-0077 Japan

**Keywords:** Giant insulinoma, Malignant insulinoma, Pancreatic neuroendocrine tumor, Ki-67 index, Grade

## Abstract

**Background:**

Larger insulinomas are reportedly more likely to be malignant; however, their biological behavior has not been clearly elucidated. We here report the characteristics and treatment of a giant insulinoma with local invasion and lymph node metastasis. We also review published reports concerning the clinical features of giant insulinomas and comparing their grading with that of pancreatic neuroendocrine tumors.

**Case presentation:**

A 71-year-old man was referred to our hospital for investigation of persistent hypoglycemia. On the current presentation, laboratory tests showed serum glucose, immunoreactive insulin, and C peptide concentrations of 45 mg/dL, 17.2 μIU/mL and 4.1 ng/mL, respectively. Dynamic magnetic resonance imaging showed a hypervascular tumor measuring 13.5 cm in the head of the pancreas. Computed tomography scanning demonstrated local invasion and lymph node involvement. He thus had Whipple’s triad, which is associated with malignant insulinoma. No distant metastases having been identified, pancreaticoduodenectomy was performed. Intraoperatively, three separate tumors were identified in the pancreatic head. Pathological examination showed all three tumors were pancreatic neuroendocrine tumors; the tumor cells in the largest mass were strongly immunoreactive for insulin. The Ki-67 index was 2–5% in most parts of the largest tumor and over 20% in the poorly differentiated areas. This tumor was classified as neuroendocrine carcinoma in accordance with the 2010 World Health Organization classification of pancreatic endocrine neoplasms. He remains free of evidence of recurrence 2 years postsurgery.

A review of published reports indicated that giant insulinomas are more malignant than smaller ones, and metastatic disease is found on presentation in 56% of patients with giant insulinomas; however, we were unable to identify any correlation between grade of pancreatic neuroendocrine tumor and biological behavior of giant insulinomas.

**Conclusions:**

Giant insulinomas more frequently exhibit malignant behavior, such as local invasion, lymph node involvement, and liver metastasis, than smaller ones. However, there was no relationship between grade and rate of metastases or survival in this small case series. Identification of useful biological markers is necessary.

## Background

Insulinomas are the commonest functioning pancreatic neuroendocrine tumor (pNET); their estimated annual incidence being one to three cases per million [1]. Approximately 10% are multiple and approximately 5% are associated with multiple endocrine neoplasia type 1 (MEN1) syndrome [2]. Their mean size is 1.5 cm; 24% are smaller than 1 cm, 42% 1–2 cm, 30% 2–3 cm, and approximately 4% >3 cm [3]. Malignancy is defined by the presence of metastases, most commonly in lymph nodes or the liver; the clinical presentation characteristically does not enable differentiating benign from malignant disease [4]. Clinicopathological evidence of malignant behavior (gross local invasion or metastases) is extremely rare in insulinomas, occurring in only 5–10% of all insulinomas; thus, most can be surgically cured [2].

Larger insulinomas are more likely to be malignant [5]; however, their biological behavior has not been clearly elucidated. Some authors have defined “giant” insulinomas as ≥9 cm in largest dimension [6, 7]. We here report the characteristics and treatment of a giant insulinoma with peripancreatic local invasion and lymph node metastasis and present a review of published reports.

## Case presentation

A 71-year-old man was referred to our hospital for investigation of hypoglycemia. Two years before, he had attended the emergency department with acute-onset loss of consciousness after a traffic accident. Hypoglycemia was identified as the cause; however, because his condition had quickly improved, he was not extensively investigated and was discharged without a definitive diagnosis. Since then, he had recurrent hypoglycemia associated with impaired consciousness. In the four preceding months, his serum glucose concentrations had been less than 67 mg/dL and he had been given intravenous glucose, which resulted in gradual resolution of symptoms.

On this presentation, his serum glucose, immunoreactive insulin and C peptide concentrations were 45 mg/dL, 17.2 μIU/mL, and 4.1 ng/mL, respectively. MEN-1 syndrome was excluded by normal serum intact parathyroid hormone, calcium, and prolactin concentrations. He thus had Whipple’s triad (symptoms known or likely to be caused by hypoglycemia, low serum glucose at the time of the symptoms, relief of symptoms when the glucose has increased to normal). To confirm the diagnosis of endogenous hyperinsulinemia caused by insulinoma, the patient underwent a supervised overnight fast during which he developed symptomatic hypoglycemia with serum glucose of 50 mg/dL.

**Fig. 1:**
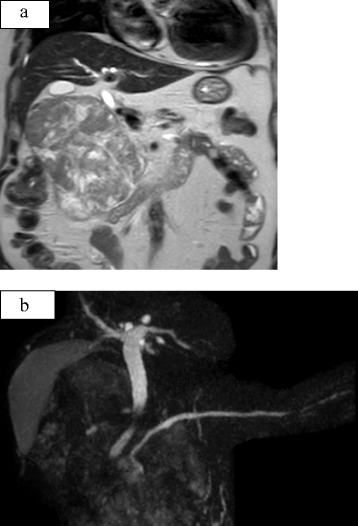
Dynamic magnetic resonance image showing a 13.5 × 12 × 8 cm hypervascular tumor containing necrotic areas in the pancreatic head (**a**). Magnetic resonance cholangiopancreatography showing no evidence of involvement of pancreatobiliary trees (**b**)

**Fig. 2:**
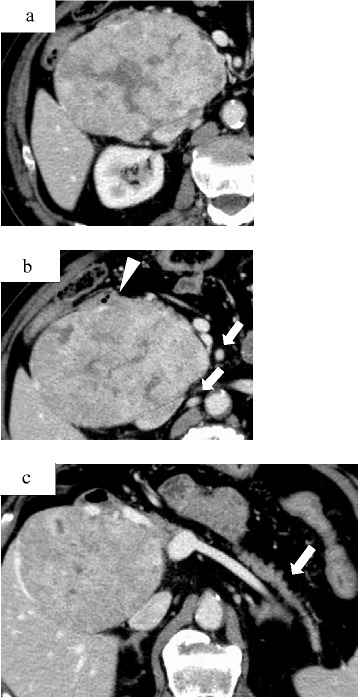
Computed tomography scan image demonstrating a heterogenous enhancing mass with areas of necrosis replacing the head of the pancreas (**a**). Computed tomography scan image demonstrating local invasion into the peripancreatic retroperitoneum and duodenum (*arrowhead*) and lymph node involvement (*arrow*) (**b**). No other hypervascular insulinomas were identified in the pancreatic body and tail (*arrow*) (**c**)

Dynamic magnetic resonance imaging showed a 13.5 cm × 12 cm × 8 cm hypervascular tumor containing necrotic areas in the pancreatic head (Fig. 1a). Magnetic resonance cholangiopancreatography showed no involvement of the pancreatobiliary systems (Fig. 1b). Computed tomography scan demonstrated a heterogenous, enhancing, partly necrotic mass partially replacing the head of the pancreas (Fig. 2a). There was no evidence of hepatic metastasis; however, local invasion of the surrounding adipose tissues and duodenum and lymph node involvement were identified (Fig. 2b). This patient’s pancreatic body and tail were slender, measuring 2 cm × 1.5 cm × 5 cm; no other tumors were identified in them (Fig. 2c). No distant metastases having been identified; extended pancreatic resection was performed.

Intraoperatively, one bulky tumor and two additional undiagnosed tumors were identified in the head of the pancreas. Manual palpation by an experienced surgeon revealed no tumor in the pancreatic body and tail. One 3.5-cm diameter pedunculated lesion was suspended from the surface of the uncinate process of the pancreas, and another 2-cm diameter tumor was attached to the duodenum. The patient underwent a subtotal stomach-preserving pancreaticoduodenectomy.

**Fig. 3:**
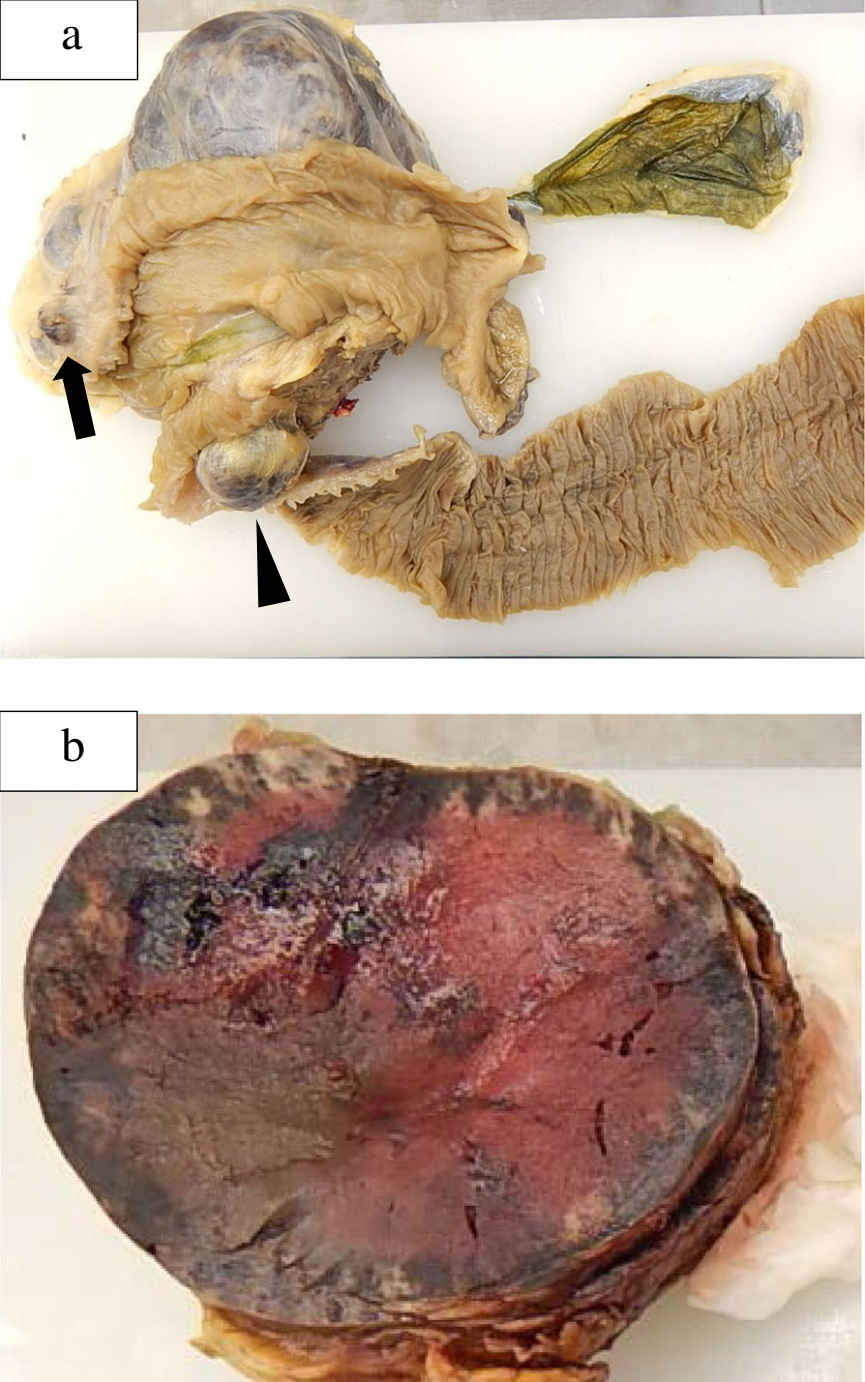
Macroscopic findings. Three separate tumors are present in the head of the pancreas, namely, the largest, which was firmly adherent to the retroperitoneum, a 2 × 2 cm tumor, which was adherent to the duodenum (*arrow*), and a 3.5 × 2.5 cm pedunculated tumor that was suspended from the surface of the uncinate process of the pancreas (*arrowhead*) (**a**). The largest mass measured 15 × 9 × 9 cm and the cut surface showed solid tissue with hemorrhagic areas (**b**)

**Fig. 4:**
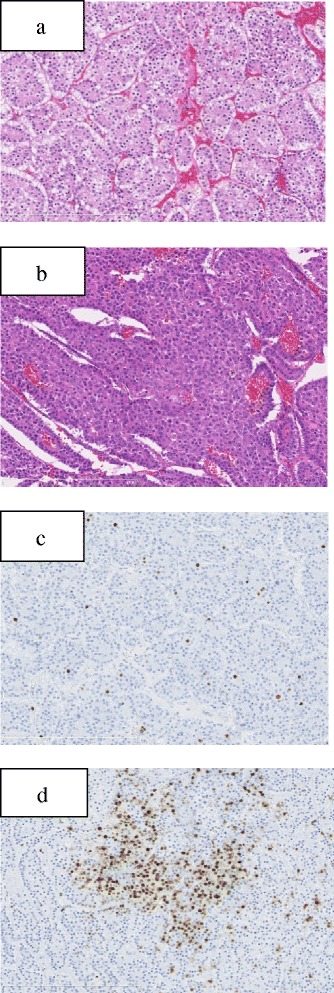
Well-differentiated tumor cells were observed in most parts of the tumor (**a**); however, poorly differentiated tumor cells were identified in a small proportion of it (**b**). The Ki67 index was 2–5% in most of the tumor (**c**); however, the poorly differentiated area had a Ki67 of over 20% (**d**)

Macroscopically, the largest tumor measured 15 × 9 × 9 cm and was firmly adherent to the retroperitoneum (Fig. 3a). Cut sections showed a solid lesion with evidence of hemorrhage (Fig. 3b). All three tumors were composed of uniform bland cuboidal cells with granular eosinophilic cytoplasm and round nuclei. Most of the largest mass comprised well-differentiated tumor cells (Fig. 4a); however, poorly differentiated tumor cells were identified in a small part of it (Fig. 4b). The retroperitoneal fat tissues and duodenum were involved, and metastases were identified in two of four peripancreatic lymph nodes. Immunohistochemically, the tumor showed strong diffuse expression of chromogranin A, insulin, and glucagon and weak expression of somatostatin. The Ki-67 index was 2–5% in most of the largest tumor (Fig. 4c) but over 20% in the poorly differentiated areas (Fig. 4d). This tumor was classified as a NEC in accordance with the 2010 World Health Organization (WHO) classification of pancreatic endocrine neoplasms.

Postoperatively, a moderate pancreatic fistula developed. This resolved with conservative treatment and he was discharged on postoperative day 35. At a routine follow-up visit 3 months postoperatively, he reported no hypoglycemic symptoms, and he remains free of evidence of recurrence 2 years and 3 months after surgery.

### Discussion

Because it has been difficult to differentiate between benign and malignant insulinomas on the basis of histologic findings, malignant insulinomas have been diagnosed based on metastasis to liver, lymph nodes, or other organs [2]. However, the new 2010 “WHO Classification of Tumors of the Digestive System” considers all insulinomas of 0.5 cm or greater malignant tumors [8]. According to the 2010 European Neuroendocrine Tumor Society guidelines, the most critical prognostic factors are the proliferative rate (mitotic index or Ki-67 labeling index) and the presence of distant metastases [9].

Surgery is the only potentially curative treatment for malignant insulinoma diagnosed at a locally advanced stage [10]. The median disease-free survival after curative resection is 5 years; recurrence occurs in more than 60% of patients at a median interval of 2.5–3 years [2]. Patients with distant metastases from insulinomas to the liver, bone, and lymph nodes have a median survival of <2 years [11].

Callacondo et al. [12] reviewed 35 cases of giant insulinoma and reported that they are more likely to be malignant than non-giant insulinomas; however, the relationship between clinical features, pNETs grading, and G1/G2/NECs has not been elucidated. In all, we reviewed 45 cases of giant insulinoma comprising the 35 cases of Callacondo et al., an additional 9 cases [4, 5, 13–19] and the present case (Table 1). We also investigated the relationship between recurrence rates and grade in reported cases (Table 2). We performed a PubMed search and Japanese MEDLINE databases (during the period of 1975–2015) using the keywords: “giant insulinoma,” “malignant insulinoma,” and “pancreatic neuroendocrine tumor” and limited our review to reports in English- and Japanese-language publications, including case reports.

**Table 1:** Clinical features of 45 reported pancreatic giant insulinomas

Variables	
Age (mean)	15–83 (57) years
Sex (M:F)	1.14:1
Tumor diameter (mean)	9–21 (12) cm
Location	
H/HB/B/BT/T/HBT/NA	3/1/2/13/22/1/3
Treatment for primary disease	
DP (+/− S)/DP+ RLM/DP+ ALM	23/2/2
PD/TP/E/TR/biopsy/NO/NA	3/1/1/4/3/5/1
Metastatic sites	
Generalized/liver/LN, surround	6/7/12
Recurrences/remnant/none	8/11/26
Recurrent sites	
Generalized/liver/local	2/4/1
Treatment of recurrences or remnant	
Surgery/Syst chemo/HACE/RT/none/NA	3/2/2/2/1/7
Outcome (*n*)	Duration of follow-up (mean) months
No disease recurrence (24)	3–276 (18)
Alive with disease (6)	15–204 (48)
Died of metastatic disease (10)	1–156 (24)
Died of other disease (1)	NA
NA (4)	NA

*H* head, *HB* head and body, *B* body, *BT* body and tail, *T* tail, *HBT* head, body and tail, *NA* not available, *DP* distal pancreatectomy, *+/−* with/without, *S* splenectomy, *RLM* resection for liver metastases, *ALM* ablation of liver metastases, *PD* pancreaticoduodenectomy, *TP* total pancreatectomy, *E* enuclation, *TR* tumor resection, *Biopsy* biopsy of pancreatic or liver tumor, *NO* no operation, *Generalized* involvement of the liver and one or more of the following organs: adrenal glands, spleen, stomach, and colon, *LN* lymph node, *Surround* surrounding tissue, *Remnant* remnant disease, *Syst chemo* systemic chemotherapy, *HACE* hepatic arterial chemoembolization, *RT* radiation therapy

**Table 2:** Clinicopathological features and grade of giant insulinomas in reports published since 2001

Author [ref]/year	Age	Sex	Size (cm)	Location	Surgery	Ki-67 (%)	Mitosis	Differentiation	Grade	Meta site at diagnosis	Recurrences	Follow-up (months)
Konno [14]/2001	40	F	10.5	Tail	DP, S	<2	NA	NA	1	None	None	NDR (24)
Mittendorf [6]/2005	65	F	9	Tail	Tumor resection	>2	<1	W	2	None	None	NDR (6)
Esteban [12]/2008	57	F	10	Tail	DP	20	NA	W	NEC	None	None	NDR (31)
Rega [12]/2009	60	M	15	Body/tail	DP, S	5.4	NA	WDEC	2	Surround, LN	None	NDR (3)
Sugiyama [7]/2010	50	M	12	Head	PD	1–2	1	W	1	Surround	None	NDR (12)
Matkari [12]/2010	32	M	11	Head	E	>2	NA	W	2	None	None	NDR (12)
Pramodh [12]/2010	81	M	9.8	NA	NO	<5	NA	WDEC	2	None	None	DOD (NA)
Oberheim [21]/2011	58	F	13.5	Head/body	PpPD	<1	1	W	1	None	None	NDR (12)
Callacondo [12]/2013	67	F	15	Tail	DP, S, RLM	<1	<1	W	1	Liver	Liver	AD (60)
Callacondo [12]/2013	63	M	10	Tail	DP, S	<2	5	W	1	LN	Liver	NDR (43)
Callacondo [12]/2013	38	M	11	Tail	DP, S, RLM	<2	3	W	2	Spleen, liver	Liver	DMD (156)
Ielpo [13]/2013	57	F	14	Tail	DP	>20	NA	Por	NEC	NA	None	NDR (72)
Eguchi [16]/2013	73	F	12	Tail	DP, S	NA	NA	NA	1	None	NA	NDR (4)
Fenech [17]/2013	76	F	16	Tail	DP, S	NA	<2	W	1	Surround	None	NA
Present case/2014	71	M	15	Head	SSpPD	>20	>20	Por	NEC	Surround, LN, duodenum	None	NDR (24)
Karavias [5]/2015	75	F	17	Body/tail	DP, S, RMD	NA	10	NA	2	Lt-kidney, Lt adrenal, PALN, liver, omentum	None	NDR (60)
Vasikasin [18]/2016	15	M	12.5	Tail	DP, S	1–2	3–4	NA	1	None	None	NDR (6)
Martino [19]/2016	49	F	21	Head/body/tail	Debulking	>15	15	NA	2	Surround, LN, liver, axillary, and mediastinal	Remnant	AD (36)

*Ki-67* Ki-67 index, *Mitosis* mitoses per 10 high-power fields, *Meta Site* metastatic sites, *DP* distal pancreatectomy, *S* splenectomy, *NA* not available, *W* well-differentiated neuroendocrine tumor, *NDR* no disease recurrence, *NEC* neuroendocrine carcinoma, *WDEC* well-differentiated neuroendocrine carcinoma, *Surround* surrounding adipose tissue, *LN* lymph node, *PD* pancreaticoduodenectomy, *E* enuclation, *NO* no operation, *DOD* died of other disease, *PpPD* pylorous-preserving PD, *RLM* resection of liver metastases, *AD* alive with disease, *DMD* died of metastatic disease, *Por* poorly differentiated neuroendocrine tumor, *SSpPD* subtotal stomach-preserving PD, *PALN* para-aorta LN, *Debulking* total pancreatectomy with splenectomy, partial gastrectomy, and cholecystectomy, *RMD* resection of metastatic diseases, *Remnant* remnant liver, axillary, and mediastinal metastases

The mean age at presentation of the 45 patients with giant insulinoma was 57 years (range, 15–83 years), and there was a slight male preponderance (male/female ratio, 1.14:1). Those patterns are similar to those of non-giant insulinomas.

Tumor location within the pancreas was available for 42 patients. Tumors in a single segment of the pancreas had a predilection for the tail (*n* = 22); involvement of both the body and tail was seen in 12 patients. Giant tumors occur more frequently in the body and tail of the pancreas because they can grow freely there without causing mass effects or mechanical obstruction [12]. Even huge masses in the head of the pancreas rarely present with gastric outlet obstruction or obstructive jaundice because insulinomas generally enlarge slowly and do not massively invade adjacent vital organs.

Surgical resection had been performed in 36 (81.8%) of all cases studied; the procedures comprising distal pancreatectomy (combined with resection or ablation of hepatic metastases) in 23 (4) cases, pancreaticoduodenectomy in 3, tumor resection in 5, and total pancreatectomy in 1 case. No patient had undergone combined resection and reconstruction of the portal vein or hepatic artery. Complete resection should be performed; however, removing 90% of the tumor is acceptable. Surgery is justified for functional advanced neuroendocrine tumors, the aims being to control symptoms, improve quality of life, and extend survival rate compared with conservative treatment [19]. Seven of 19 patients with metastases or recurrence had received sequential multimodal therapy (systemic chemotherapy, chemoembolization, ablation, and resection of liver metastases). These aggressive therapies can prolong the survival of patients with malignant insulinoma, even in the presence of liver metastases [20].

The diameter of these patient’s tumors ranged between 9 and 21 cm (median, 12 cm). Most insulinomas become symptomatic when very small, allowing early detection and prompt surgical treatment, possibly before they can metastasize. The severity of hypoglycemia varies from patient to patient and has no direct relationship with tumor burden [10]. Sugiyama et al. [7] suggested that giant insulinoma initially diagnosed as non-functioning pNETs may secondarily become functioning.

Twenty-five (55.6%) of the cases of giant insulinoma reviewed had metastases at presentation, this percentage being higher than the 10% reported in most insulinoma series [2]. At the first surgery, six patients had involvement of the multiple organs, seven had liver metastases only, and 12 had locally advanced disease such as invasion of the surrounding adipose tissues or/and lymph node involvement. Eight cases had developed recurrences, the major site of metastases being the liver.

Follow-up data were available for 41 of the published cases. Duration of follow-up of 24 cases who had no recurrences ranged between 3 months and 23 years (median 18 months). Seven cases of the 11 fatal cases were reported before 1980. The overall survival of all patients who died with disease ranged between 1 month and 13 years (median, 2 years), whereas patients of non-giant malignant insulinoma with distant metastases had a median survival of <2 years [11]. Six cases alive with disease had been followed-up for 15 months to 17 years (median, 4 years).

Since 2001, the mitotic activity, Ki-67 index, and grade have been reported for 17 patients with giant insulinomas (Table 2) [5–7, 12–14, 16–19, 21]. We compared the grade, the rate of metastases and duration of follow-up of 18 cases, including the present case. The Ki-67 index was <2% in eight cases; the only three cases with a Ki-67 index of ≥20% were diagnosed as having NECs. Four, three, and one patient had no metastases from G1/G2/NEC at surgery, respectively, whereas four, four, and one had synchronous metastases, respectively. Unexpectedly, there was no difference in proportion of G1/G2/NEC in patients with versus without synchronous metastases. The median durations of follow-up by grade were as follows: 12 months (G1), 12 months (G2), and 31 months (NEC); the duration of follow-up for NECs being longer than expected. We anticipated that patients with giant insulinoma and higher Ki-67 indexes would have worse prognoses; however, we identified no relationship between grade and survival. In our small case series, we were unable to identify any correlation between grade and biological behavior of giant insulinomas.

## Conclusions

Giant insulinomas more frequently exhibit malignant behavior, such as local invasion, lymph node involvement, and liver metastasis, than smaller ones. Metastatic disease is present at surgery in 56% of cases of giant insulinoma. Unexpectedly, there was no relationship between grade of pNET and rate of metastases or survival in this small case series. Identification of useful biological markers is necessary, as is development of anti-tumor agents for liver metastases.
